# Investigation of respirable particulate matter pollutants on air-breathing zone workers in the Beam Rolling Mills Factory (Iran National Steel Industrial Group), Ahvaz, Iran

**DOI:** 10.4103/0019-5278.43264

**Published:** 2008-08

**Authors:** Masoud Rafiei, Alaka S. Gadgil, Vikram S. Ghole, Neemat Jaafarzadeh, Sharad D. Gore, Mohammad Aberomand, Mitra Shabab

**Affiliations:** Department of Occupational Hygiene, Imam Hospital, Ahvaz Jundishapour University of Medical Sciences, Ahvaz, Iran; 1 Environmental Science, University of Pune, Pune - 411 007, India; 2 Chemistry, Division of Biochemistry, University of Pune, Pune - 411 007, India; 3 Statistics, University of Pune, Pune - 411 007, India; 4 Dept. of Environmental Health, Imam Hospital, Ahvaz Jundishapour University of Medical Sciences, Ahvaz, Iran - 6193673166; 5 Biochemistry, Imam Hospital, Ahvaz Jundishapour University of Medical Sciences, Ahvaz, Iran - 6193673166; 6 Clinical Research, Imam Hospital, Ahvaz Jundishapour University of Medical Sciences, Ahvaz, Iran - 6193673166

**Keywords:** Beam Rolling Mills, pollutants, respirable particulate matter, workplace

## Abstract

Workers of iron and steel factories are exposed to a wide range of pollutants depending on the particular process, the materials involved, the effectiveness of monitoring and the control measures. Adverse effects are determined by the physical state and propensities of the pollutant involved, the intensity and duration of the exposure, the extent of pollutant accumulation in the body and the sensitivity of the individual to its effects. The main aim of this study is to assess the levels of the indoor respirable particulate matter (RPM) and to compare the health condition of exposed workers, with nonexposed employees group. Line 630 has only one furnace of 40 tons and line 650 has two furnaces of 20 and 40 tons capacity due to which the mean of the RPM concentrations in the breathing zone was significantly different (*P* < 0.05) in line 650 but not in line 630 as compared with National Institute for Occupational Safety and Hygiene's (3 mg/m^3^). The average of the RPM concentrations in production line 650 is higher than that of production line 630, with the 95% confidence interval in saw cabin station number 1 of production line 650.

## INTRODUCTION

Air pollution from iron- and steel-making operations has always been an environmental concern. These pollutants, including particulate matters (PMs) such as soot and dust that may contain iron oxides, have been the focus of controls. Some effects are immediate while others may take years and even decades to develop. Changes in processes and equipments along with improvement in measures to keep exposures below toxic levels have reduced the risks to the workers. However, these have also introduced new combinations of pollutants and there is always a danger of accidents, fires and explosions.[1,2]

**Figure 1:**
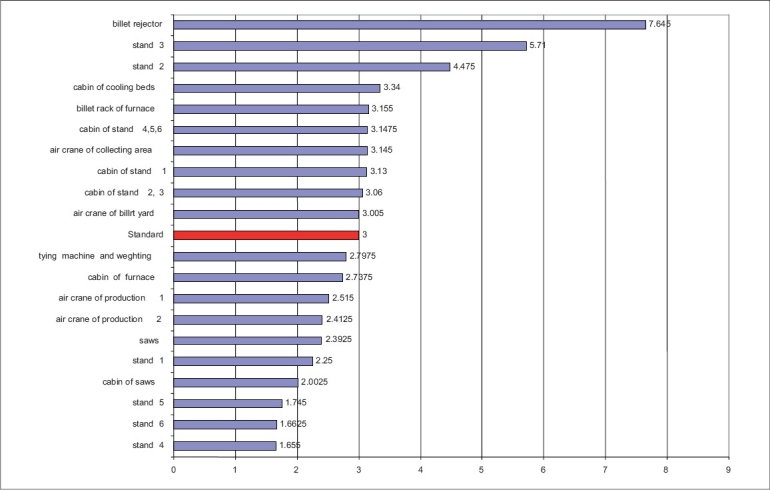
Comparison of mean value of RPM concentrations with NIOSH standard for 8 hrs. In Line 630

In order to evaluate the indoor air pollution as well as the health of the workers, a cross-sectional project was conducted in Beam Rolling Mills Factory, Ahvaz-Iran, 2003-2005. The Beam Rolling Mills Factory has two production lines: 630 and 650, with a nominal capacity of 190 000 and 195 000 tons/year, respectively, and is capable of producing various types of beams. There are two lines: 630 and 650, and both of them are similar. However, there are differences in calibration of grindings and number of stands. The diameter of grinding for stand one in line 630 is 630 mm and that of line 650 is 650 mm. There is only one furnace with a capacity of 40 tons in line 630, whereas in line 650 there are two furnaces with a capacity of 20 and 40 tons. Production line 630 has a physical area of 650 m^2^, which is more than line 650. In line 650, there are six stands (rolling machines), while in line 630 there are five stands. In line 650, stands 1, 2, 3, 4, 5, 6 and 7 carry out five, two, three and one pass, whereas in line 630, stands 1, 2, 3, 4, 5 and 6 carry out five, three, three and one pass, respectively [Figure 1]. The process of production lines 630 and 650 are illustrated as follows:[3]

Because the process of iron and steel production consumes energy, fuel, raw materials (i.e., steel billets, etc.) and water therefore this process leads to the production and emission of airborne pollutants throughout the workplace and threatens the health of the workers. In other words, due to the existence of air conditioners in these plants, they can be regarded as an additional reason for the emission of air pollutants into the outdoor. As a result, they threaten the health of the inhabitants who live around these plants. This causes direct and indirect damages to humans, plants and animals. Epidemiological studies have shown that PM air pollution is associated with cardiovascular mortality and morbidity, especially particles with aerodynamic diameters under 2.5 µm (PM_2.5_). Recent studies have shown an association between PM pollution and autonomic functions, including heart rate (HR), blood pressure and HR variability. However, the association and linking mechanisms have not been clearly demonstrated in animal studies.[4,5]

## MATERIALS AND METHODS

Stratified sampling has been used to draw the samples, each station being considered as a stratum with proportional allocation. The National Institute for Occupational Safety and Hygiene (NIOSH) no. 550 was used for the determining the RPM concentrations in the Beam Rolling Mills Factory. Sampling schedule was dependent on the process of production in the Beam Rolling Mills Factory. In a period of 8 h, four samples of RPM were taken from each sampling station.[6]

Production lines 630 and 650 have been divided into 20 stations. The RPM is considered in indoor production lines of the location of RPM sampling stations.[7]

Calibration of flow rate pump was carried out with an electronic bubble meter (Dry cal DC-Lit Bios, SKC Model, London, England) and a curved line of calibration was obtained (0.178 + 0.768X). During sampling from the workers’ breathing area, climatic parameters (temperature, relative humidity and air pressure) were recorded. Samples were collected using a low-volume sampling pump (SKC Model, London, England) operated at a flow rate of 2 l/min-1 on membrane filters with a pore size 0.5 µm and a diameter of 37 mm.

In production line 650, the number of RPM concentrations was 20 samples with the number of replications being four times in a duration of 8 h. In line 630, the number of RPM concentrations was 20 samples with the number of replications being four times for 0the same duration.[6]

The RPM was determined using an analytical balance with 0.01 mg precision e=0.001gr, d=o.oo1gr. Hague, Switzerland.

## RESULTS

As shown in Table 1, the mean concentrations of RPM in production line 650 were significantly different from the NIOSH standard (vis. 3 mg/m^3^, *P* < 0.05 *t*-test).

**Table 1:** Comparison of the Mean Value of RPM* Concentrations of Separation in Lines 630 and 650 with the NIOSH Standard for 8 h in terms of mg/m^3^

Source	Sampling size	Mean	Standard deviation	*P*-value	95% CI	
					
					Lower	Upper
Line 630	80	3.09	± 1.43	0.76	2.52	3.85
Line 650	80	3.78	± 1.03	0.03	3.48	5.04

* Respiratory particulate matter (RPM) *P* < 0.05; The National Institute for Occupational Safety and Hygiene (NIOSH) standard = 3 mg/m^3^; Statistical method (*t*- test); Confidence interval (CI)

**Figure 2:**
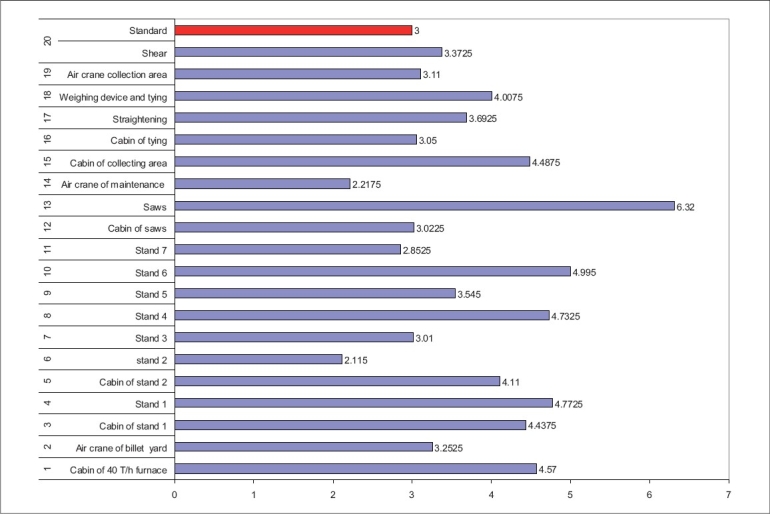
Comparison of mean value RPM concentrations with NIOSH standard for 8 Hours in Line 650

**Figure 3:**
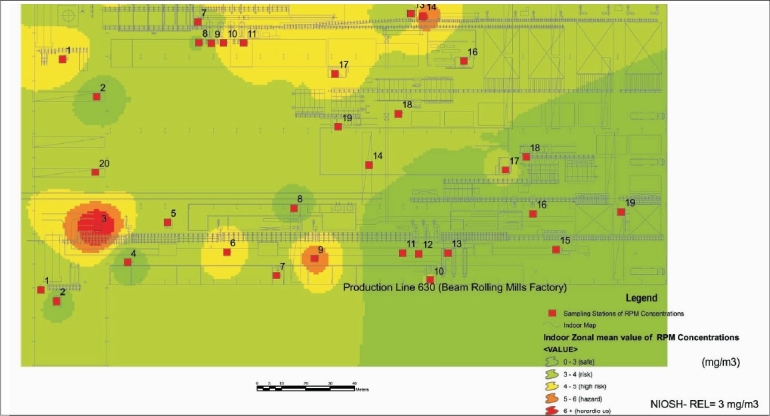
Map 1 location evaluation mean value of RPM concentrations in lines 630 and 650

The maximum RPM concentrations [Figures 2 and 3] are from the saw station cabin shown in line 630 (7.64 mg/m^3^) and the minimum are from the four stations in line 650 (1.65 mg/m^3^).

## DISCUSSION

There are significant differences between the mean value of RPM concentrations of separation in lines 630 and 650 and the NIOSH standard [Table 1].

There are differences in calibrations and number of stands in lines 630 and 650. In final mills actions in line 650, there are four working stands and saw machines, whereas in line 630, there are only three working stands and one saw machine. As a result, the average of the RPM concentrations in line 650 (3.78 mg/m^3^) is higher than that of line 630 (3.09 mg/m^3^), with 95% confidence interval. In saw cabin station number 1 of line 650 (6.37 mg/m^3^), a maximum concentration was found because of four saw machines used for iron cutting and were located around station 13. A high speed of the saws generates a high air velocity causing air turbulence, due to which the dust does not settle fast. Saw cabins are located above saws that are air conditioned. The inlet fan sucks the air with a high RPM and pushes it into the cabin. Therefore, particulate pollutants are released into the work environment. In Figure 2, it was illustrated that there is a maximum RPM concentration in the Billet rejector station in line 630 (7.64 mg/m^3^), as station Billet rejector carries out the peel action on metal and primary stands. Stand one carries out five passes on the soft metal that makes primary beams. As this station is located near the door of the billet storage and transport place, the pollution load in this station is more than in the other stations. The above stations are near the melting furnace of line 630. Production Processing results in emission of some PMs Differences between lines 630 and 650 are as follows:

1. The distance among stands in line 650 is less than that of line 630.
2. The area of production in line 650 is one third of the area of line 630 (650 m^2^).
3. Production line 650 is in the vicinity of the Rod and Mills Factory.
4. Production line 650 is in the proximity of the main road and there are light and heavy transports.
5. The maximum concentration of RPM is in the saws cabin because line 650 produces beams with sizes 14 and 18 mm, while line 630 produces beams of 10 and 12 mm.
6. Light and heavy transport with the discharge of waste is observed.

Table 2 and Figure 3, map no. 1 in line 630 show 50, 35, 5, 5 and 5% mean value of concentrations in comparison with the NIOSH standards i.e., 10, seven, one, one and one stations were at safe, risk, hazard and hazardous levels, respectively.

**Table 2:** Location and evaluation of the mean value of respirable particulate matter concentrations in line 630

Location	No. of stations	%
Safety level	10	50
Risk level	7	35
High-risk level	1	5
Hazard level	1	5
Hazardous	1	5
Total	20	100

Table 3 and Figure 3, map no.1 for line 650 illustrate 10% mean value of concentrations, i.e., two stations were at safe level, 45% (nine stations) were at risk level, 30% (six stations) were at high-risk level and 15% (three stations) were in hazard level.[8]

**Table 3:** Location and evaluation of the mean value of respirable particulate matter concentrations in line 650

Location	No. of stations	%
Safety level	2	10
High-risk level	6	30
Hazard level	3	15
Hazardous	-	-
Total	20	100

Therefore, production line 650 has a higher risk than production line 630.

## References

[1] Kiely P, Yap D, De Brou GB, Fraser D, Dong W (1997). A comparative study of Toronto's air quality and selected world cities. Presented at the 90^th^ air and waste management association.

[2] (1999). Airborne Particles Expert group, Source Apportionment of Airborne Particulate Matter in the United Kingdom.

[3] Iran National Steel Industrial Group (INSIG). Brochure literature.

[4] Chang TY, Jain RM, Wang CS, Chan CC (2003). Effects of occupational noise exposure on blood pressure. J Occup Environ Med.

[5] Cedex C (2007). Air pollutant emissions prediction by process modelling: Application in the iron and steel industry in the case of a re-heating furnace. Hand books of NIOSH manual of analytical methods.

[6] NIOSH (1997). Particulates not otherwise regulated, respirable, NIOSH Manual of Analytical Methods (NMAM). NIOSH of the US. Dept. of Health and Human Services of the United States of the American.

[7] Mdermott HJ (2005). Pumps and flow rate calibraration: Air monitoring for toxic exposures.

[8] Herlow M (2004). Rhonada Pfaff. Arc GIS 9 using Arc Map.

